# The Importance of Writing and Publishing Case Reports During Medical Training

**DOI:** 10.7759/cureus.1964

**Published:** 2017-12-19

**Authors:** Christian Ortega-Loubon, Carlos Culquichicón, Ricardo Correa

**Affiliations:** 1 Cardiac Surgery, Clinic University Hospital of Valladolid; 2 Emerge, Emerging Diseases and Climate Change Research Unit, School of Public Health and Administration, Universidad Peruana Cayetano Heredia; 3 Division of Endocrinology, Diabetes and Metabolism, The Warren Alpert Medical School of Brown University

**Keywords:** medical education, case report, medical writing, scientific writing, medical publishing

## Abstract

Case reports are valuable resources of unusual information that may lead to new research and advances in clinical practice. Many journals and medical databases recognize the time-honored importance of case reports as a valuable source of new ideas and information in clinical medicine. There are published editorials available on the continued importance of open-access case reports in our modern information-flowing world. Writing case reports is an academic duty with an artistic element. Unfortunately, few physicians-in-training receive formal education on what constitutes a publishable case report. This article emphasizes that the medical education community, specially the graduate medical education community, should be aware of the importance of writing and publishing good quality case reports.

## Editorial

Introduction

Improvements to patient outcomes can result from important studies, investigations, and advances in clinical practice that have been conducted as a result of valuable information and new ideas presented in case reports, which are published today by many journals [1]. The importance of having access to case reports in this emerging, demanding, and changing modern world continues to be recognized [2]. In 1920, Osler, one of the fathers of modern medicine, stated: “Always note and record the unusual … Publish it. Save it on a permanent record as a short, concise note. Such communications are always of value.”

The value of case reports may be underestimated compared to other publications that are more detailed and supported by evidence, but the contribution of case reports has been remarkable. In fact, sharing such valuable and trustworthy information has led to discoveries and the introduction of treatments for novel diseases [3]. This experience-based informative method has been converted into an accepted academic form of publication with the ability to spread knowledge quickly to a wide medical professional audience.

A work of art

Writing case reports is an academic duty with an artistic element as the authors are required to communicate the clinical findings in a simple scientific manner. This equilibrium of artistic and research skills can be attained only with a constant publication of case reports. Academic wisdom, clinical skills, and creative writing all help to attract the attention of an editor to publish a report and the readers to read it [3]. Case report writing should be a fusion of academic knowledge, logical thinking, and the creative skill of attracting readers’ attention or making colleagues want to know more about a specific case report [4]. Unfortunately, few junior physicians receive formal training on what constitutes a publishable case report. Physicians are initially exposed to case reports during medical school, and many may write their first case report as a resident under the mentorship of one of their attending physicians.

Broad recommendations exist concerning how and when to write a case report and its framework. While journals have established acceptance criteria, such as the content and format of a publication, case reports are not standardized and their quality can be variable [4]. Guidelines are available for different publication types, for example, observational studies (Strengthening the Reporting of Observational Studies in Epidemiology), systematic reviews and meta-analyses (Preferred Reporting Items for Systematic Reviews and Meta-Analyses), and randomised controlled trials (Consolidated Standards of Reporting Trials). Guidelines are also available for adverse event case reports, and all authors should be familiar with the Committee on Publication Ethics and the Enhancing the Quality and Transparency of Health Research Network. However, there were no guidelines for case reports until September 2013, when the international reporting guidelines for case reports (CARE) was created [2].

Lessons learned

Some barriers we have had to face when dealing with case reports were: lack of implementation of guidelines for writing case reports, restricted access to bibliographic resources in developing countries, mentors with little enthusiasm for publishing scientific papers, work overload, medical training that focused only on the academic aspects neglecting the scientific aspects, language barriers that made it difficult to publish in journals with a high impact factor, and financial barriers to publish in open access journals. After experiencing these issues several times in writing and publishing case reports, we have learned the importance of having a strict methodology to ensure success.

A possible solution may be a major change in training on the scientific aspects: teaching the importance of writing case reports from the early stages of a medical career, even when we are medical students because it offers an excellent opportunity to gain experience in scientific writing. The practice of continuous writing will help physicians-in-training to develop the skills necessary to make any paper worth publishing, by sharpening their writing abilities and providing critical peer review experience.

Writing at a high standard is not an easy task and can take a lot of time, but it will produce a case report that will help the medical community understand more about individualized medicine. This was reflected in our country, where after medical students started learning the basic principles of writing case reports, the scientific production of case reports increased as did their presentation at the local, regional, national, and international levels. Students improved their skills for developing and presenting case reports, and some have even won awards in international competitions. Furthermore, students gained experience of the editorial process with the submission of an increased number of case reports to local medical journals [5].

Conclusion

The medical education community should be aware of the importance of writing and publishing good quality case reports; this type of article may also be a resource for physicians to increase their publication success during their training years.
